# Use of Long Term Molecular Dynamics Simulation in Predicting Cancer Associated SNPs

**DOI:** 10.1371/journal.pcbi.1003318

**Published:** 2014-04-10

**Authors:** Ambuj Kumar, Rituraj Purohit

**Affiliations:** Bioinformatics Division, School of Bio Sciences and Technology, Vellore Institute of Technology University, Vellore, Tamil Nadu, India; University of Maryland, Baltimore, United States

## Abstract

Computational prediction of cancer associated SNPs from the large pool of SNP dataset is now being used as a tool for detecting the probable oncogenes, which are further examined in the wet lab experiments. The lack in prediction accuracy has been a major hurdle in relying on the computational results obtained by implementing multiple tools, platforms and algorithms for cancer associated SNP prediction. Our result obtained from the initial computational compilations suggests the strong chance of Aurora-A G325W mutation (rs11539196) to cause hepatocellular carcinoma. The implementation of molecular dynamics simulation (MDS) approaches has significantly aided in raising the prediction accuracy of these results, but measuring the difference in the convergence time of mutant protein structures has been a challenging task while setting the simulation timescale. The convergence time of most of the protein structures may vary from 10 ns to 100 ns or more, depending upon its size. Thus, in this work we have implemented 200 ns of MDS to aid the final results obtained from computational SNP prediction technique. The MDS results have significantly explained the atomic alteration related with the mutant protein and are useful in elaborating the change in structural conformations coupled with the computationally predicted cancer associated mutation. With further advancements in the computational techniques, it will become much easier to predict such mutations with higher accuracy level.

## Author Summary

The genetic mutations in human were shown by single nucleotide polymorphisms (SNPs), and several of them were supposed to cause deleterious and disease associated phenotypic effects. The ability to differentiate between pathogenic and neutral nsSNPs (non-synonymous Single Nucleotide Polymorphisms) using computational approaches could significantly help in targeting the disease associated mutations. In this work we applied computational tools to prioritize the most deleterious disease associated mutation in Aurora kinases. We used sequence and structural based approaches to refine cancer associated mutation and applied a long term simulation (MDS) to understand the changes in structural conformation and function of the aurora kinases upon mutation. From the 200 ns MD simulation, we suggested that the G325W mutation induced major phenotypic damages in aurora-A kinase protein and altered its structural behaviour in 3D space, which might play an important role in inducing hepatocellular carcinoma.

## Introduction

Most genetic variations in human were portrayed by single nucleotide polymorphisms (SNPs), and most of them were thought to cause phenotypic differences amid individuals. Due to the use of high-throughput sequencing methods, the amount of identified variants in the human genome was rising rapidly, but studying their phenotypic outcome was a laborious task. Moreover, processing of these vast amounts of SNPs needs the improvement of regular annotation tools. Detecting pathological genetic variants were helpful in the organization of genome level study, establishing the hope on the way of target based therapies and tailored medicine. The ability to differentiate between pathogenic and neutral nsSNPs (non-synonymous Single Nucleotide Polymorphisms) using computational approaches could significantly aid in targeting the disease associated mutations. In last two decades, extensive computational efforts had been provided to study the functional and structural consequences of the SNPs [1]. The primary efforts to recognize the patterns of SNPs taking place in the coding regions of genes were studied by Cargill et al. [2] and Halushka et al. [3]. Cargill et al. characterized SNPs in 106 candidate genes having potential relevance to various human diseases [2]. Several reports had very precisely explained the occurrence and consequences of disease-associated alleles on the protein structures. Terp et al. [4] reported the structurally relevant features common in disease-associated mutations from the Human Gene Mutation Database (HGMD). Vitkup et al. [5] examined the frequency of mutations associated with diseases in detail. Furthermore, the detailed analysis of the biophysical and evolutionary distributions of the disease-associated mutations was demonstrated by Ferrer-Costa et al. [6]. Stitziel et al. [7] further analysed the locations of non-synonymous disease-associated polymorphisms by organizing the mutational data into structural classes. Moreover, Mooney et al. [8] demonstrated the majority of occurrence of disease-associated mutations in the highly conserved genomic locations. Furthermore, Saunders and Baker [9] applied decision trees and a linear logistic regression to find that a protein structure-derived solvent accessibility term (C*β* density) and an evolutionary term derived from a PSSM matrix (SIFT) to examine different features associated with intolerant mutations. Moreover, Krishnan and Westhead [10] applied decision tree and support vector machine (SVM) techniques to the in vitro mutagenesis data sets as well as to SNPs in the nematode worm species *Caenorhabditis elegans* to examine their effectiveness in SNP characterization. These efforts were further accompanied by several other researches that demonstrated the effective use of computational tools and algorithms for accurate characterization of SNPs [1]. Path to deleterious SNP prediction has now advanced from its previous form. Apart from reporting the deleterious nsSNPs, now the calculation of phenotypic changes had further raised the prediction accuracy level. The previous computational SNP analysis pathways lack the genotype-phenotype correlations which were considered as the major disadvantage of the result. To overcome these limitations, our lab studied the structural consequences of deleterious predicted mutations. Changes in amino-acid composition had wide role in affecting the native conformation of the protein structure.

The vibrant nature of proteins was closely linked to its function, with key conformational changes usually taking place on timescales, varying from microseconds to several seconds. At a scrupulous time step, protein attains particular conformation that occupies a least amount on its free-energy landscape. When energy fluctuates from one minimum to another, it leads to alterations in the protein structure which controls their structural fluctuations and function. Thus to examine these conformational alteration occurring due to predicted deleterious alleles, we further incorporated molecular dynamics calculations for native and mutant protein structures. Molecular dynamics (MD) simulation is an absolute method for modelling protein dynamic motions, the classification of which gives perception into the workings of macromolecular systems at spatial and temporal scales that are hard to access empirically. The use of molecular dynamics approach had been a leading platform to determine the phenotypic consequences induced by point mutations [11]–[14]. Our previous results had shown high accuracy of deleterious nsSNP prediction in CENPE and MCAK protein coding genes when MDS (Molecular Dynamics Simulation) was incorporated [15], [16]. On the technical side, depending on the size of the protein, the simulation might take a very long time to converge (e.g. a hundred nanoseconds). Thus, even the most ‘state-of-the’ art or computationally intensive methods might not give correct results. Moreover, folding was typically much slower than what presently simulated through direct application of all-atom MD simulations. Most simulation studies of protein folding had relied on reduced models, implicit solvent models, protein unfolding or the inclusion of experimental data into the simulations [17]. Extended MD simulations were liable to play a vital role in modelling the long-timescale native and mutant state dynamics and their association with protein function. Thus, we carried out a long term molecular dynamics simulation to aid the computational SNP prediction results. Aurora kinases were chosen to detect the cancer associated SNPs due to the recent cases showing their involvement in cancer associated pathways.

## Materials and Methods

### Dataset collection

Human Aurora kinase protein sequence data was collected form national centre for biological Information (NCBI) protein sequence database [18]. SNP information for our computational analysis was obtained from NCBI dbSNP (http://www.ncbi.nlm.nih.gov/snp/) [19]. Structure of Aurora-A kinase was obtained from Brookhaven Protein Data Bank [20] (PDB ID: 1MQ4). The mutant structure was build using homology modelling technique through Modeller9v9 package [21]. Modelled structures were refined by means of loop refining, checking wrong bond contacts and adding hydrogen atoms. The best selected structure was energy minimized by charmm27 force field for 5000 iterations using Gromacs 4.5.3 package [22].

### Disease-associated SNP prediction

There are likely chances of amino acid variants to cause pathological phenotype, which might directly or indirectly lead to cancers. We used SIFT [23], Polyphen-2 [24], PhD-SNP [25], Pmut [26], MutPred [27], Dr Cancer [28], Fathmm [29] and SNP Function Portal [30] to detect the amino acid variants which are likely to cause cancer. SIFT and Polyphen-2 is used to filter deleterious and harmful SNPs from the large pool of SNP dataset. Moreover, PhD-SNP and Pmut was used to filter disease associated SNPs. Furthermore, Dr. Cancer, Fathmm and SNP Function Portal was used to predict the cancer associated SNPs. MutPred was used to examine the molecular changes occurring in the protein induced by a particular amino acid variant.

### Phenotype analysis

SNPeffect4.0 tool was used to detect the phenotypic changes induced by the computationally predicted cancer associated mutation [31]. It reports the changes in TANGO, LIMBO, WALTZ and ddG scores for the given mutation. Furthermore, the meta-analysis tool of SNPeffect4.0 used to map these changes for different cancer type.

### Cation-π sites and stabilizing residues

Cation-π interactions were calculated by using CaPTURE program [32]. The CaPTURE program identifies energetically significant cation–π interactions within proteins in the Protein Data Bank (PDB). Cation–π interactions in Aurora_A kinase structures are evaluated with default parameters which were mentioned in our previous work [33].

### Identifying stabilizing residues

SRIDE tool was used to identify the stabilizing residues in Aurora-A protein [16], [34]. The prediction is done by calculating 4 essential criteria that involves surrounding hydrophobicity of a residue, the long-range order, stabilization centre defined by considering the conservation scores of residues and the contact map of a protein [16]. Two residues are in contact if there is at least one pair of heavy atoms with a distance less than the sum of the van der Waals radii of the two atoms plus 1.0 Å [16]. A contact is measured long-range if it is between residues that are separated by at least 10 residues in the protein sequence [16]. Two residues are stabilizing centers (SC) elements if they are involved in long-range contacts and if at least one supporting residue can be found in each of the flanking tetra-peptides of these residues, in such a way that at least seven out of the possible nine interactions are formed between the two triplets [16]. Combinedly, all these criteria are compiled to report the stabilizing residues [16].

### Molecular docking analysis

We used molecular docking analysis to carry comparative analysis between TPX2 binding affinity of native and mutant Aurora A protein using HADDOCK tool [35], [36]. HADDOCK is information driven flexible docking approach to perform the molecular docking simulation and it runs a series of scripts in combination with ARIA [37], [38] and CNS [39]. The interacting active residues of Aurora-A and TPX2 for docking simulations were obtained from our previous work [40].

### Molecular dynamics simulation

Molecular Dynamics Simulation was carried out by using Gromacs 4.5.3 package [22]. Structure of native and mutant Aurora-A kinase was used as starting point for MD simulations. The simulation parameters were set according to our previous work conducted on Aurora-A protein [40] and other proteins [15], [32], [40]. Systems were solvated in a rectangular box with TIP3P water molecules at 10 Å marginal radius. The systems were neutralized by added 3 sodium ions (Na^+^) to the simulation box using the “genion” tool that accompanies with Gromacs package. Energy minimization was performed for 5000 iterations by conjugate gradient method implementing GROMOS96 43a1 force field. Emtol convergence criterion was set to 1000 kJ/mol/nm. Berendsen temperature coupling method [41] was used to control the temperature within the box. Electrostatic interactions were calculated using the Particle Mesh Ewald method [42]. The systems were subjected to position restraint simulation for 5 ns and after that unrestraint simulation for 200 ns. We then computed the comparative analysis of structural deviations in native and mutant structure. RMSD, RMSF, SAS and Rg analysis were carried out by using g_rms, g_rmsf, g_sas and g_gyrate tool respectively. Number of distinct hydrogen bonds (NHbonds) was calculated using g_hbond. Moreover, we implemented g_densmap to obtain the atomic density distribution of the native and mutant protein. All graphs were plotted using Grace GUI toolkit 5.1.22 version. Further we carried out principal component analysis using essential dynamics (ED) method according to protocol [43] within the Gromacs software package. This section is an abbreviated version of our previously published work [15], [32], [40].

## Results/Discussion

**Figure 1:**
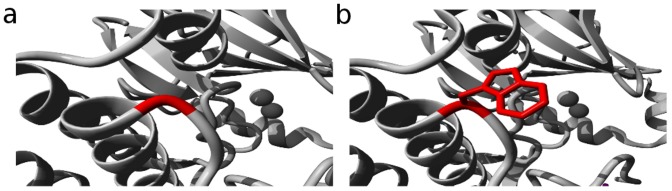
Location of the mutant residue. a) Native Aurora-A protein b) Mutant Aurora-A protein. The mutant location is shown in red.

Total 60 nsSNPs were computationally examined to detect their harmful and damaging properties. Out of 60 SNPs, 24 were calculated to be deleterious as well as damaging using SIFT and Polyphen2 servers (Table 1). Among these 24 nsSNPs, 7 were accounted to be exceedingly deleterious and damaging with tolerance index of zero and PSIC score 1. To further categorize the expected deleterious nsSNP's as cancer-associated, we used PhD-SNP, Pmut, MutPred, Dr Cancer, Fathmm and SNP Function Portal. Combinedly we reported Aurora-A G325W mutation as cancer-associated (Table S1). A very interesting scenario was observed in the results obtained from SNP Function Portal. Aurora-A G325W mutation was predicted to be associated with multiple cancer cases including Neurofibrosarcoma, Prostate cancer, Colorectal cancer, Breast cancer, Li-Fraumeni syndrome, Osteosarcoma, Hepatocellular carcinoma, Histiocytoma, Nasopharyngeal carcinoma, Thyroid carcinoma, Pancreatic cancer, Breast cancer, Adrenal cortical carcinoma, Gastric cancer, Endometrial carcinoma, Colorectal cancer, Lung and Ovarian Adenocarcinoma. This large number of cancer-associated predictions directly suggested that the G325W Aurora-A kinase mutation was likely to be associated with cancer cases. Fig. 1 showed the position of mutant residue in Aurora-A kinase protein.

**Table 1:** Deleterious and damaging nsSNPs prioritized using SIFT tolerance score and Polyphen-2 PSIC score.

Gene	SNP ID	AA change	SIFT		Polyphen-2	
			Tolerance Index	Prediction	PSIC	Prediction
**AurkA**	rs113451501	N395S	0.94	Tolerated	0.000	Benign
	**rs146034224**	**P381L**	**0.00**	**Deleterious**	**0.994**	**Damaging**
	rs33923703	M373V	0.01	Deleterious	0.000	Benign
	rs192771776	H366Y	0.50	Tolerated	0.019	Benign
	rs201356620	E354Q	0.12	Tolerated	0.699	Damaging
	rs45557632	F348L	0.01	Deleterious	0.353	Benign
	rs200181472	R343Q	0.15	Tolerated	0.063	Benign
	**rs11539196**	**G325W**	**0.00**	**Deleterious**	**1.000**	**Damaging**
	**rs45483697**	**G198S**	**0.00**	**Deleterious**	**1.000**	**Damaging**
	**rs45520831**	**R179K**	**0.03**	**Deleterious**	**0.903**	**Damaging**
	rs45455492	L114Q	0.66	Tolerated	0.001	Benign
	rs2230743	S104L	0.30	Tolerated	0.005	Benign
	rs45533839	V84A	0.76	Tolerated	0.000	Benign
	rs1047972	I57V	1.00	Tolerated	0.000	Benign
	rs34572020	P50L	0.27	Tolerated	0.027	Benign
	rs145616804	Q34H	0.43	Tolerated	0.975	Damaging
	**rs188825988**	**R24C**	**0.00**	**Deleterious**	**1.000**	**Damaging**
	rs6069717	G11R	0.87	Tolerated	0.003	Benign
**AurkB**	rs201709756	A344T	0.07	Tolerated	0.012	Benign
	**rs148715809**	**P325S**	**0.03**	**Deleterious**	**0.933**	**Damaging**
	rs144169786	S313L	0.74	Tolerated	0.068	Benign
	rs146017427	S313P	0.30	Tolerated	0.019	Benign
	**rs146334050**	**I304T**	**0.00**	**Deleterious**	**0.989**	**Damaging**
	rs1059476	M298T	0.42	Tolerated	0.000	Benign
	rs151173438	A294T	0.26	Tolerated	0.006	Benign
	**rs140224531**	**R284C**	**0.00**	**Deleterious**	**0.998**	**Damaging**
	**rs149651741**	**G212R**	**0.00**	**Deleterious**	**0.945**	**Damaging**
	**rs146905713**	**E204D**	**0.00**	**Deleterious**	**0.975**	**Damaging**
	**rs55871613**	**T179M**	**0.02**	**Deleterious**	**0.989**	**Damaging**
	**rs199630207**	**E174K**	**0.00**	**Deleterious**	**0.970**	**Damaging**
	**rs147097910**	**A157T**	**0.00**	**Deleterious**	**1.000**	**Damaging**
	rs148133660	R147W	0.01	Deleterious	0.121	Benign
	**rs150216235**	**V103M**	**0.04**	**Deleterious**	**0.997**	**Damaging**
	rs3027254	H100Q	0.13	Tolerated	0.001	Benign
	**rs184713921**	**R95Q**	**0.00**	**Deleterious**	**1.000**	**Damaging**
	rs141907099	R70W	0.00	Deleterious	0.292	Benign
	rs146036524	T69M	0.14	Tolerated	0.000	Benign
	rs199981964	S61G	0.45	Tolerated	0.001	Benign
	rs74385486	M58I	0.19	Tolerated	0.000	Benign
	rs55878091	A52V	0.33	Tolerated	0.000	Benign
	rs201438176	R44H	0.05	Tolerated	0.886	Damaging
**AurkC**	rs200296015	M1T	0.05	Deleterious	0.012	Benign
	rs200712786	S12N	0.43	Tolerated	0.003	Benign
	rs146186252	R17Q	0.06	Tolerated	0.205	Benign
	**rs137858773**	**R28H**	**0.00**	**Deleterious**	**0.603**	**Damaging**
	rs61736320	I60V	0.03	Deleterious	0.001	Benign
	**rs148631645**	**I60T**	**0.00**	**Deleterious**	**0.617**	**Damaging**
	rs202030166	N83H	0.20	Tolerated	0.955	Damaging
	**rs45555141**	**R86H**	**0.00**	**Deleterious**	**0.862**	**Damaging**
	**rs45623632**	**Y90C**	**0.00**	**Deleterious**	**0.983**	**Damaging**
	**rs199855150**	**H92R**	**0.01**	**Deleterious**	**0.928**	**Damaging**
	**rs45503793**	**T126M**	**0.02**	**Deleterious**	**0.996**	**Damaging**
	**rs147955649**	**C138F**	**0.00**	**Deleterious**	**1.000**	**Damaging**
	rs200042694	K178N	0.58	Tolerated	0.001	Benign
	**rs199933542**	**D202Y**	**0.00**	**Deleterious**	**1.000**	**Damaging**
	rs149157434	S222N	0.08	Tolerated	0.089	Benign
	**rs45527835**	**D236Y**	**0.03**	**Deleterious**	**0.889**	**Damaging**
	rs147392532	R238K	1.00	Tolerated	0.000	Benign
	rs201199082	Q267E	0.93	Tolerated	0.001	Benign
	rs200445619	Q267R	0.54	Tolerated	0.006	Benign

The alleles highlighted in bold are predicted to be deleterious as well as damaging by SIFT and Polyphen scores.

**Figure 2:**
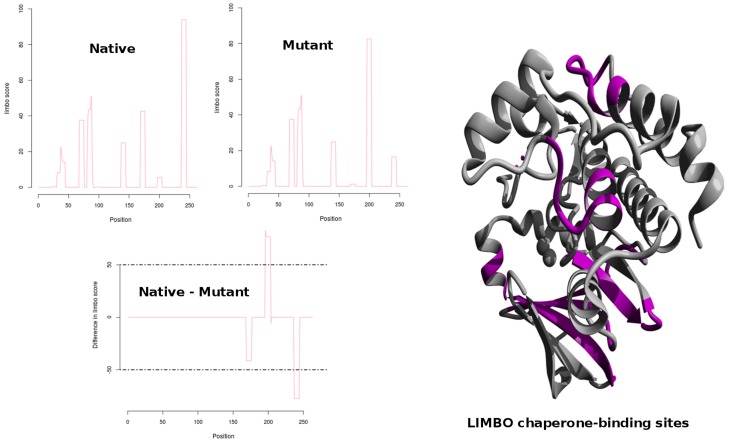
Profile representation of the LIMBO stretches in a) Native and b) Mutant protein. c) Difference in LIMBO chaperone binding propensity between native and mutant. d) Molecular visualisation of LIMBO chaperone-binding sites as pink colored segments.

**Figure 3:**
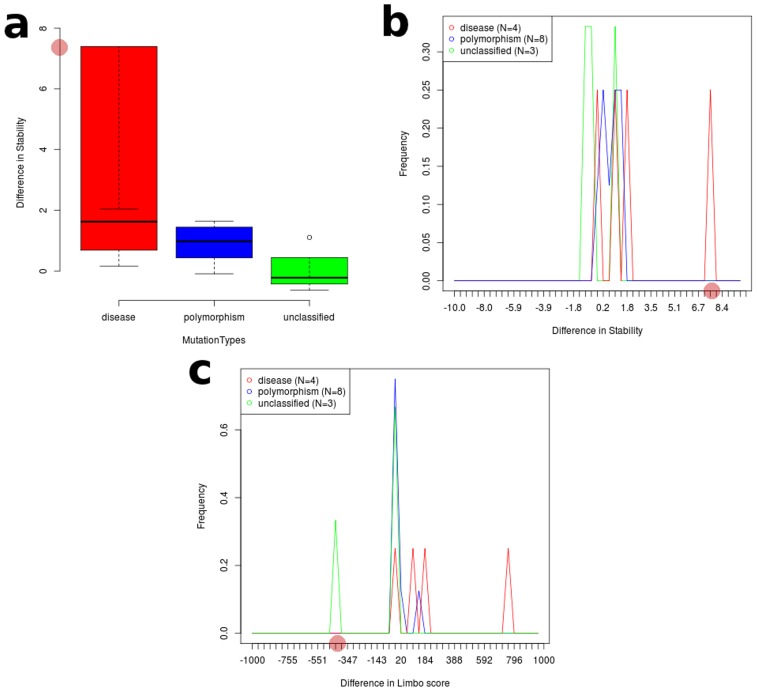
Representation of dLIMBO and ddG scores for the mutations associated with Hepatocellular carcinoma. a) Difference in stability scores b) Difference in stability scores with respect to its frequency of occurrence. c) Difference in LIMBO scores with repect to its frequency of occurrence.

Without studying the phenotypic evidences associated with a particular mutation, the conclusion derived from computational studies are not easily reliable. Thus we implemented SNPEffect4.0 to examine the phenotypic consequences of G325W mutation at molecular level. Significant fluctuation in the LIMBO score for G325W mutation was observed. For G325W mutation, dLIMBO equaled −335.67 which meant that the mutation decreased the chaperone binding propensity of the protein. Fig. 2 represents the position of the chaperone-binding sites in the protein. In Fig 2, it could be clearly seen that the mutation had shifted the chaperone-binding sites from the 7^th^ stretch to the 6^th^ stretch. In table 2, we could see that the 6^th^ stretch had shown significant rise in the chaperone-binding tendency where in native, the LIMBO score of 6^th^ stretch was 5.66 whereas in mutant the LIMBO score increased to 82.59. Furthermore the 7^th^ stretch showed significant loss in the chaperone-binding tendency in mutant when compared to the native. Moreover, the 5^th^ chaperone-binding stretch was completely absent in the mutant protein, whereas it was shown to be active in native Aurora-A kinase. Molecular chaperones are protein molecules that support in the non-covalent folding or unfolding and the assembly or disassembly of other biomolecular structures. Furthermore, it disallowed both newly synthesised polypeptide chains and assembled subunits from aggregating into non-functional structures. Decrease in chaperone-binding tendency of Aurora-A might severely damage the folding pattern of the protein. Moreover, it would lead to the formation of non-functional protein aggregates which might cause disease-associated phenomena. Furthermore, major stability loss was observed in the case of mutant when FoldX predictions from SNPEffect4.0 were taken into account. The mutant showed rise in ddG value of 7.28 kcal/mol. This implied that the mutation severely reduced the protein stability. Based on the dLIMBO and ddG scores it was evident that the mutation had induced loss in the chaperone-binding tendency of protein which further led to severe conformational changes in the protein motif and reduced its stability. We subjected these scores for the meta-analysis in order to detect the type of cancer which was most likely to occur in the case of G325A Aurora-A kinase mutation. The dLIMBO and ddG scores were plotted for previously reported cancer cases. Our dLIMBO and ddG scores were shown to lie in the dLIMBO and ddG distribution range of hepatocellular carcinoma associated mutations (Fig. 3). For other cancer cases, no positive distributions for the above obtained scores were observed. This showed that the G325W mutation might play an important role in inducing hepatocellular carcinoma associated phenotypes, whereas no significant correlation was observed for other cancer cases. Other scores obtained from SNPEffect4.0 did not have much significance.

**Table 2:** LIMBO regions in mutant and wild type.

Number	Start	End	Stretch	Score
**Native**				
1	30	44	KFILALKVLFKAQL	12.87
2	67	75	ILRLYGYF	37.58
3	80	89	VYLILEYAP	40.52
4	136	144	LLLGSAGE	24.91
**5**	**168**	**176**	**DYLPPEMI**	**42.67**
**6**	**196**	**204**	**FLVGKPPF**	**5.66**
**7**	**236**	**244**	**RLLKHNPS**	**94.00**
**Mutant**				
1	30	44	KFILALKVLFKAQL	12.87
2	67	75	ILRLYGYF	37.58
3	80	89	VYLILEYAP	40.52
4	136	144	LLLGSAGE	24.91
**5**	**—**	**—**	**—**	**—**
**6**	**195**	**203**	**EFLVWKPP**	**82.59**
**7**	**236**	**244**	**RLLKHNPS**	**16.53**

For each LIMBO region, the start, end, sequence and score is given. Stretches shown in bold represents the LIMBO regions that have been severely affected by the mutation.

By the interaction with TPX2, the catalytic activity of Aurora-A protein increased up to 15-fold [44]. TPX2 bound to and localized Aurora-A to spindle microtubules along with microtubules on the periphery of spindle poles [45]. To examine the changes in interaction pattern of mutant Aurora-A with TPX2 when compared to the native, we conducted molecular docking analysis. In-depth analysis of the docked complexes revealed notable differences between interaction patterns of native and mutant Aurora-A protein with TPX2. As we described in [14], computation of interaction energy was very important to comprehend the attraction level of biological partners. Overall interaction energy amid Aurora-A (native/mutant) and TPX2 protein mainly contributed to their electrostatic and van der Waals interaction energy. In native, there were a major contribution of electrostatic and van der Waals energy of −97.7+/−24.1 kcal/mol and −41.0+/−5.6 kcal/mol respectively (Table 3). The total protein–protein interaction energy of native complex was −153.17 kcal/mol (Table 3). All the results obtained from docking analysis were confirmed from the results obtained in our previous work on Aurora-A kinase [40]. Electrostatic and van der Waals energy confirms large amount of surface complementarities amid native Aurora-A and TPX2. On the contrary, mutant complex showed less van der Waals and electrostatic energies of −17.9+/−2.1 and −59.3+/−14.8 kcal/mol, respectively, and relatively less amount of total interaction energy −92.96 kcal/mol when compared to the native (Table 3). More negative value of interaction energy for native as compared to mutant indicated its high affinity towards TPX2. Approximations of van der Waals interaction energy were computed to provide a theoretical quantitative estimation of the protein–protein non-bonded interactions [14]. Result showed that the mutation induced damaging consequences in native conformation of Aurora-A kinase that in turn reduced its overall binding affinity to TPX2 protein.

**Table 3:** Statistical analysis of protein–protein docking result obtained by HADDOCK.

Structure	HADDOCK score	Total Interaction energy (Kcal mol^−1^)	Van der Waals energy (Kcal mol^−1^)	Electrostatic energy (Kcal mol^−1^)	Desolvation energy (Kcal mol^−1^)	Restraints violation energy (Kcal mol^−1^)	Buried surface area (Å^2^)
Narive Complex	−74.7+/−7.7	−153.17	−41.0+/−5.6	−97.7+/−24.1	−29.4+/−6.9	151.4+/−3.96	1201.6+/−32.9
Mutant Complex	−37.3+/−4.9	−92.96	−17.9+/−2.1	−59.3+/−14.8	−11.7+/−6.1	60.6+/−2.64	758.5+/−28.8

As we described in [14], buried surface area (BSA) is a criterion to assess protein surface which is not exposed to water and more BSA showed a compact macromolecular complex. In table 3, we have showed the BSA of native and mutant complexes as 1201.6+/−32.9 and 758.5+/−28.8 respectively. Consideration of desolvation component (the loss of interactions with the water phase) was important because it overcompensated the interaction energy and resulted in an opposite effect [14], [20]. Restraints violation energy, desolvation energy and BSA showed good correlation with interaction energy and docking score of complex during docking simulation (Table 3). Major difference between docking score, interaction energy and BSA were found in mutant than native. The net values of native were significantly higher than mutant, indicating the loss-of-function due to mutation in Aurora A which disrupted the binding with TPX2 when compared to mutant (Table 3). Docking process solely depended on the position of binding residues (active residues) on the protein surface. It showed that divergence from the actual location of binding residues (position number 155, 157, 159 and 197) in mutant Aurora-A might be a reason for the loss of binding with TPX2. The three factors namely, score difference in docking process, alteration in interaction energy and in buried surface area of complex possibly corresponded to conformational variation of protein-binding surface due to mutation.

**Figure 4:**
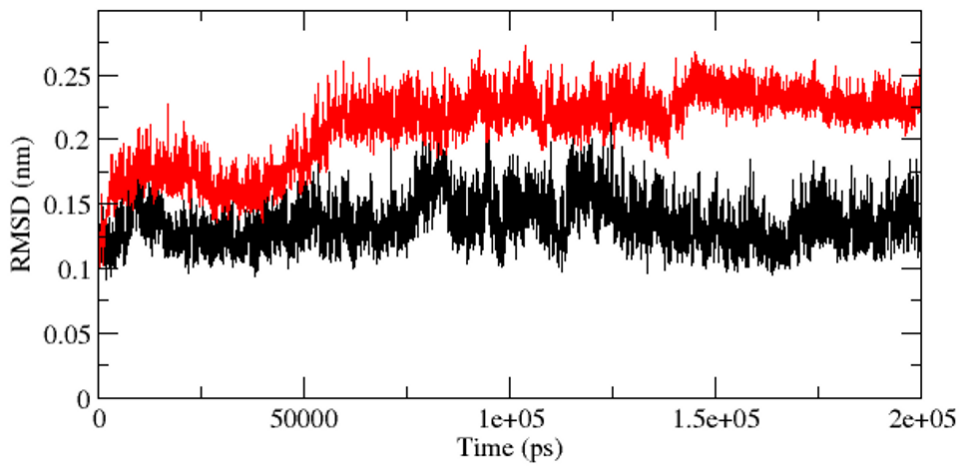
Backbone RMSDs are shown as a function of time for native and mutant Aurora-A protein structures at 300 K. Native is shown in black and mutant in red.

**Figure 5:**
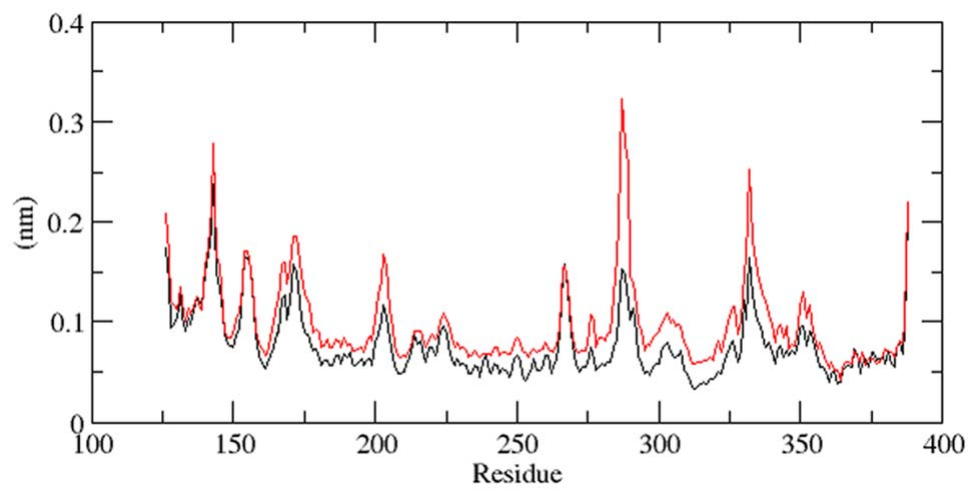
RMSF of the backbone CAs of Cα atoms of native and mutant Aurora-A protein versus time at 300 K. Native is shown in black and mutant in red.

The ddG scores obtained from SNPeffect4.0 FoldX prediction implicated loss in the stability of protein. Thus we implemented MDS calculation to observe the molecular alterations associated with the stability loss. We explored RMSD, Rg, RMSF, SASA, NHbond, total energy and density distribution of mutant and native structure. RMSD for all the Cα atoms were calculated from the starting structure which was described as the central origin to compute the protein system (Fig. 4) [46]. In Fig. 4, native and mutant protein showed diverge fashion of deviation from starting, resulting in final backbone RMSD of ∼0.172 nm and 0.252 nm respectively during the simulation. Mutant lingered distinguished till the end and exhibited higher aberration than native. This level of fluctuation together with a minute difference in average RMSD value after the relaxation time led to the outcome that the simulation produced stable trajectory, thus providing a suitable basis for further investigation. To observe the affect of mutation on the dynamic behaviour of residues, the RMSF values of mutant and native backbone residues were computed (Fig. 5). Inspection of fluctuation score exposed the occurrence of higher degree of flexibility in mutant. Mutant curve differed significantly and fluctuated at higher degree during the simulation time period, indicating that mutant conformation was flexible throughout the simulation time and its structure acquired expanded conformation when compared to native.

**Figure 6:**
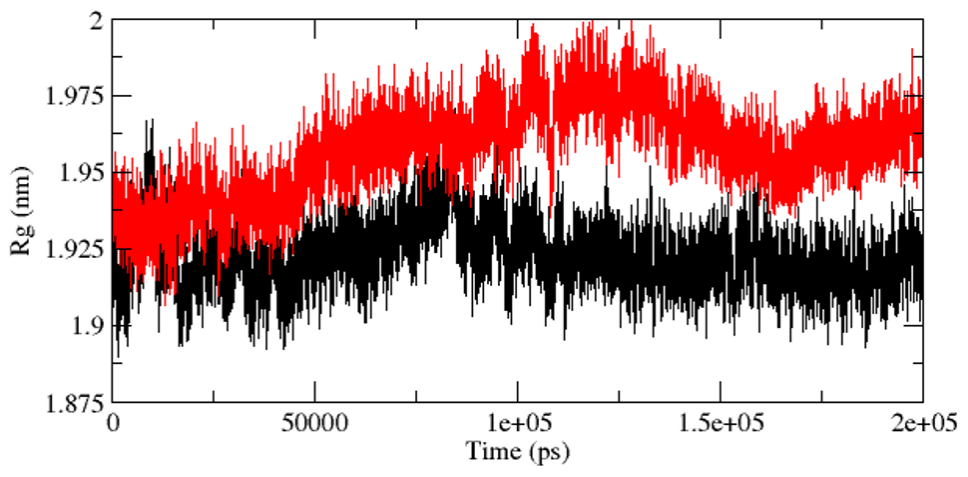
Radius of gyration of Cα atoms of native and mutant Aurora-A protein versus time at 300 K. Native is shown in black and mutant in red.

**Figure 7:**
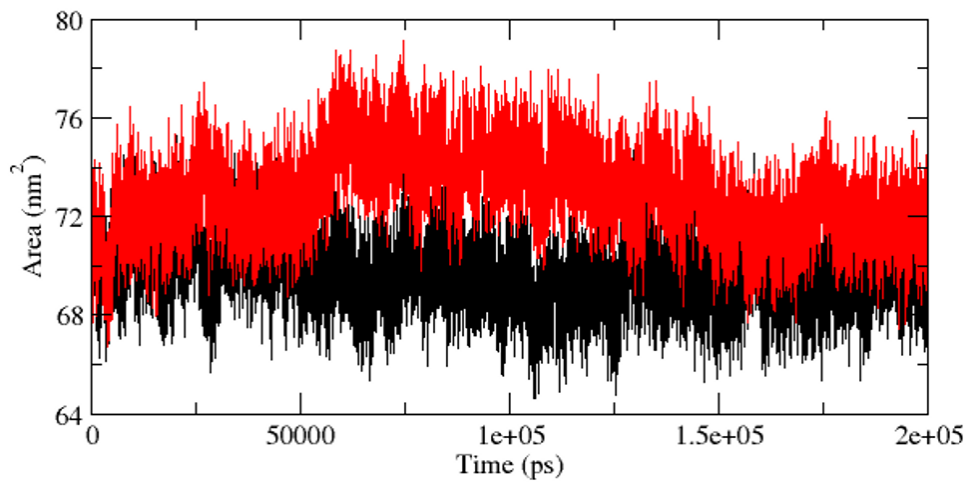
Solvent-accessible surface area (SASA) of native and mutant Aurora-A protein versus time at 300 K. Native is shown in black and mutant in red.

**Figure 8:**
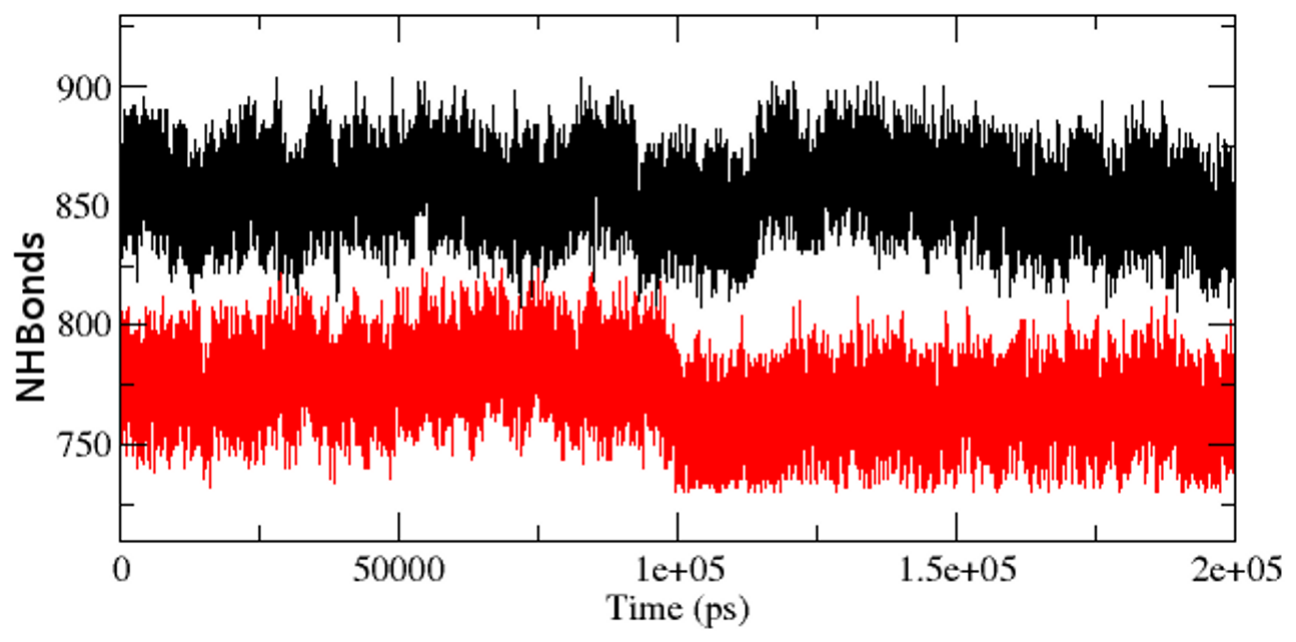
Average number of protein–solvent intermolecular hydrogen bonds in native and mutant Aurora-A protein versus time at 300 K. Native is shown in black and mutant in red.

**Figure 9:**
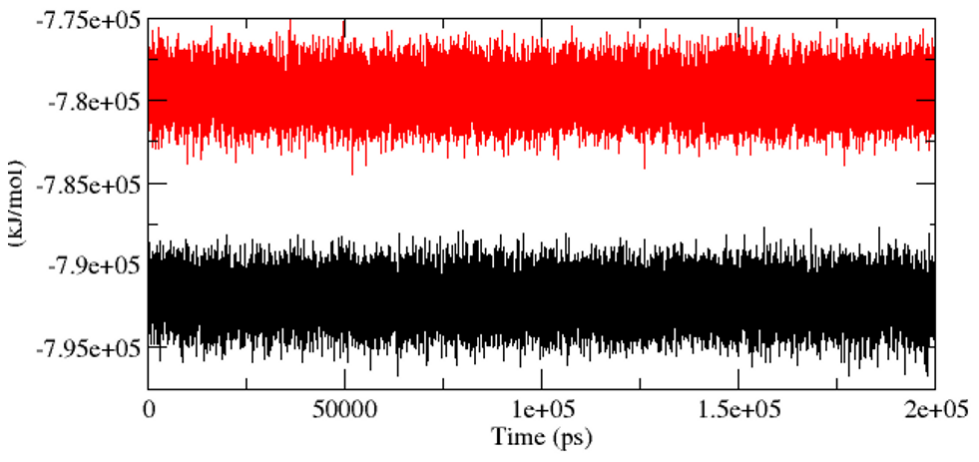
Graph of total energy as a function of time for mutant and native Aurora A protein versus time at 300 K. Mutant is shown in red and native in black.

**Figure 10:**
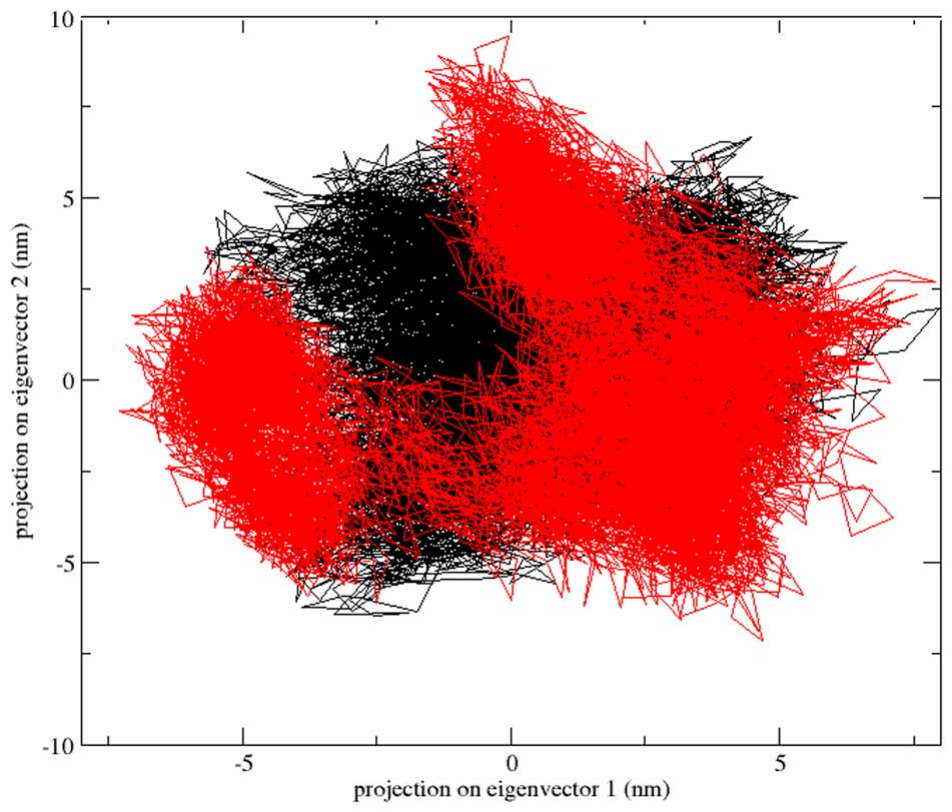
Projection of the motion of the protein in phase space along the first two principal eigenvectors at 300 K. Native is shown in black and mutant in red.

As we described in [46], the radius of gyration (Rg) is the mass-weighted root mean square distance of group of atoms from their common centre of mass. Hence it provided an observation into global dimension of protein. Radius of gyration graph for alpha-carbon atoms of protein vs time at 300 K is depicted in Fig. 6. We observed a major fluctuation in both native and mutant between a time periods of 0 to 200 ns (Fig. 6). Based on Rg graph, native structure was found stable than mutant. The alteration in SASA of mutant and native protein with time is depicted in Fig. 7. Mutant protein showed higher value of SASA with time, while native indicated lower SASA value. Longer variation in Rg plot showed that the mutant protein might be enduring a major structural transition. This was further maintained by SASA result in which the mutant was found to reveal higher values (Fig. 7). Hydrogen bond plays a major factor on controlling the steady conformation of protein. Time dependent NHbond formations during simulation were observed in native and mutant proteins in order to comprehend the relationship between hydrogen bond formation and flexibility. Mutant structure demonstrated significantly less number of NHbond formation throughout the simulation when compared to native (Fig. 8). Furthermore, the energy spectrum provided a detail insight into the stability of a particular structure. We investigated the overall energy variation of native and mutant protein throughout the simulation. A significant rise in energy level of mutant structure was observed (Fig. 9). It provided a clear indication of loss of the stability in mutant structure. Moreover, the observation of corresponding eigenvalues showed the level of variation and dynamic nature of protein molecule in the simulation system and was mostly restricted within the first two eigenvectors. Our result showed higher range of eigenvector trajectory covered by mutant in comparison with native (Fig. 10).

**Figure 11:**
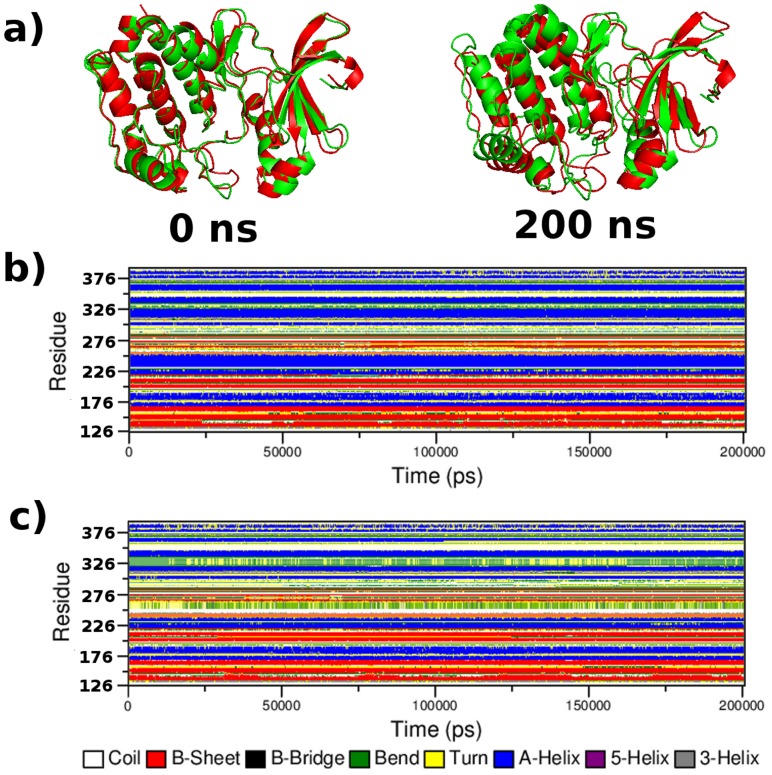
Time evolution of the secondary structural elements of the protein at 300 k (DSSP classification). a) Native and Mutant Aurora-A protein merged at different timescale. b) Native Aurora-A secondary structural variation **c**) Mutant secondary structural fluctuation.

Further information on the structural plasticity of protein was found by the analysis of time-dependent secondary structures and structural fluctuations. Fig. 11 showed the secondary structural elements as a function of simulation time. Coils, β-sheets, bends and turns were found in both native and mutant protein during simulation time period and it is depicted in fig 11. In native Aurora-A kinase, the residues between position 238–275 appeared as alpha-helix, β-sheets and coils with few bend conformation till the end of the simulation but in mutant Aurora-A this region showed beta-sheets and turns with more traces of bend conformation. The severe conformational changes in this region supported our previous results obtained from SNPEffect4.0, where the loss in chaperone-binding tendency of this region was observed. In Comparison to native Aurora-A kinase, mutant showed significant structural changes between a region of residues 238–275, 324–334 and 348–360 during simulation. Between residues 324–334, native showed more α-helix whereas in mutant it attained bend with few traces of turns. Between the residues of 348–360, α-helix and coil conformation formed the majority of structure whereas in mutant, the coil conformation was only seen. In other regions, very slight changes in secondary structure conformation were observed.

**Figure 12:**
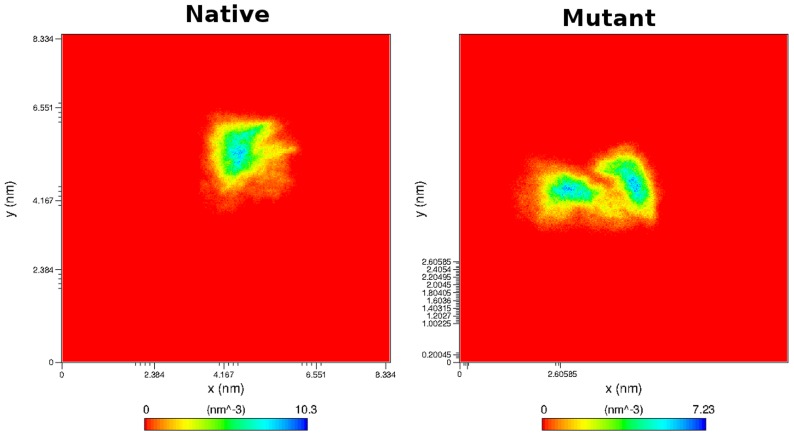
Density distribution of 7^th^ chaperone-binding stretch in a) native and b) mutant Aurora kinase A.

**Figure 13:**
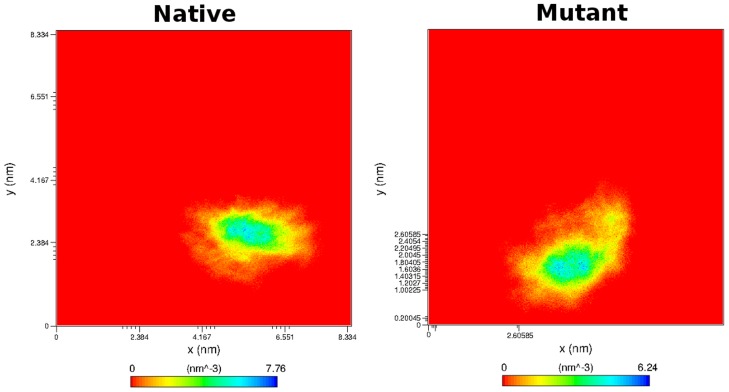
Density distribution of 5^th^ chaperone-binding stretch in a) native and b) mutant Aurora kinase A.

In SNPeffect4.0 results, we observed that the chaperone-binding location shifted from the 7^th^ stretch to the 6^th^ stretch. Moreover, the chaperone-binding activity of 5^th^ stretch was shown to be completely lost. Thus we plotted the atom density distribution to check if these shifting had caused any major changes in the orientation and atomic distribution of 7^th^ and 5^th^ chaperone-binding stretches. The plot indicated major conformation loss in mutant as its atomic distribution differed significantly from the native. For the 5^th^ stretch the mutant structure confirmed a highest atomic density of 6.24 nm^−3^ whereas in native it was 7.76 nm^−3^ (Fig. 12). Moreover, for the 7^th^ stretch, the mutant structure exhibited a highest atomic density of 7.23 nm^−3^ whereas in native it was 10.3 nm^−3^ (Fig 13), which was in accordance to the DSSP results, further indicating major conformational loss in these segments.

**Figure 14:**
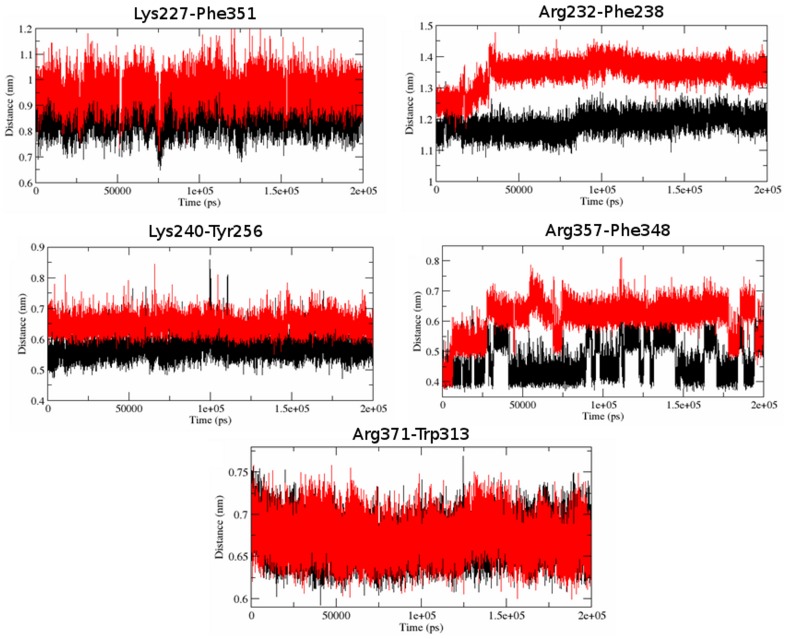
Distance fluctuation between the cation-π residues a) Lys227-Phe351 b) Arg232-Phe238 c) Lys240-Tyr256 d) Arg357-Phe348 e) Arg371-Trp313. Native is shown in black and mutant in red.

The above results strongly indicated the loss of stability of the mutant structure. To examine the molecular causes associated with these outcomes, we further investigated the cation- π interactions and the stabilizing residues in Aurora A protein. Cation–π interaction has significant contribution in protein stability. In proteins, cation–π interactions occur between the aromatic side chains of phenylalanine (F), tyrosine (Y), and tryptophan (W) and the cationic side chain of lysine (K) or arginine (R). It was likely to have a important role in the folding or stabilization of the proteins. The significance of cation–π interaction had been observed in several researches for their corresponding role in retaining the stability of proteins. We observed a total of 5 energetically significant cation–π interactions (Fig 14). To scrutinize if the cation–π interactions were maintained in mutant structure, we further examined the bond length variations of the outlying cation–π interaction. The bond length fluctuation showed that the Arg232-Phe238, Lys240-Tyr256 and Arg357-Phe348 cation–π interactions had undergone major interaction losses (Fig 14). After 32900 ps there was a major loss in Arg232-Phe238 cation–π interaction whereas after 28400 ps there was a significant loss in Arg357-Phe348 interactions (Fig 14). Mutant trajectory of Arg371-Trp313 bond length was comparatively higher when compared to the native and maintained a constant trajectory during the simulation. Arg232-Phe238 and Lys250-Tyr246 interaction were retained in the mutant structure. These results suggested that there was a major loss in cation–π interactions in the mutant structure which might have induced higher fluctuation and lower in stability of the structure. Furthermore, the protein structure contains certain SC that acted as an essential component to maintain the stable conformation of corresponding protein structure. Residues Ala160, Leu161, Lys162, Val163, Asn192, Leu210, Leu263, Lys271, Ile272 and Ser278 were identified as stabilizing centers. When the RMSF of these residues were investigated, we observed significant rise in their fluctuation levels (Table 4). This further indicated that apart from damaging the ATP binding domain and the cation-π interactions, the mutation had also introduced major deflection in the Aurora A protein SC, which might have significantly contributed towards phenotypic changes observed in mutant G325W structure. All these biophysical and biochemical factors obtained from 200 ns MD simulation had collectively suggested that the G325W mutation had strong evidence of inducing phenotypic damages in Aurora-A kinase protein, and might play significant role in inducing hepatocellular carcinoma. These results could be further implemented for wet lab investigations and could provide an evidence of Aurora kinase genetic mutation in association with cancer.

**Table 4:** The RMSF values of stabilizing residues.

Serial Number	Residue	HP	LRO	RMSF (nm)	
				Native	Mutant
1	ALA160	27.54	0.02299	0.0589	0.0736
2	LEU161	24.29	0.02299	0.0528	0.0657
3	LYS162	21.97	0.03065	0.0586	0.0672
4	VAL163	24.01	0.03448	0.0669	0.0849
5	ASN192	20.39	0.03065	0.0575	0.0707
6	LEU210	23.47	0.03065	0.0497	0.0664
7	LEU263	23.05	0.02299	0.0545	0.07
8	LYS271	24.49	0.02299	0.0589	0.0717
9	ILE272	21.37	0.02299	0.0507	0.0697
10	SER278	21.55	0.03448	0.0518	0.0753

### Conclusion

By the use of multiple computational platforms and long-term molecular dynamics simulation approaches, we identified Aurora-A G325W mutation as highly deleterious and associated with hepatocellular carcinoma. The RMSF, Rg, DSSP and energy fluctuation results provided a clear insight of stability loss and conformational changes in mutant protein. The results showed that the long-range computational simulations provided clear picture of conformational changes occurring in the computationally predicted disease associated mutant protein structure. Through the advancements in the computational resources, the use of MDS for higher time scale would become approachable. Dedicated computer for biomolecular simulation will most likely bring further speedups in future, allowing the length of each simulation to move well ahead of the large time*scale*. These advancements will aid in studying the changes in the dynamics behavior of computationally predicted disease-associated SNPs. Moreover, the advancements in high-performance molecular dynamics simulation of biomolecules will provide new prospects for the use of *in silico* simulations to observe mutation-induced changes in biophysicochemical properties of protein, especially for those parameters, which were well-known to occur at long timescales. Furthermore to the capacity to produce very long-range incessant simulation trajectories, these advances will also greatly enhance the accuracy of SNP predictions, ultimately allowing us to examine the changes occurring at the atomic state in a very precise manner [46].

## Supporting Information

Table S1 Cancer-associated nsSNPs predicted using PhD-SNP, Pmut, MutPred, Dr Cancer and Fathmm server. g score, P score, molecular changes and prediction were obtained from MutPred server. SEQPROF results were obtained from Dr Cancer server. Allele highlighted in bold has been predicted to show Cancer-associated SNPs.
(DOC)
