# Supplementary material for: Use of Long Term Molecular Dynamics Simulation in Predicting Cancer Associated SNPs
Source: PLoS Comput Biol. 2014 Apr 10;10(4):e1003318. doi: 10.1371/journal.pcbi.1003318 (PMC3983272; doi:10.1371/journal.pcbi.1003318)
Supplement: Table S1 — Cancer-associated nsSNPs predicted using PhD-SNP, Pmut, MutPred, Dr Cancer and Fathmm server. g score, P score, molecular changes and prediction were obtained from MutPred server. SEQPROF results were obtained from Dr Cancer server. Allele highlighted in bold has been predicted to show Cancer-associated SNPs. (DOC) [file pcbi.1003318.s001.doc]

**Table S1.** Cancer-associated nsSNPs predicted using PhD-SNP, Pmut, MutPred, Dr Cancer and Fathmm server. g score, P score, molecular changes and prediction were obtained from MutPred server. SEQPROF results were obtained from Dr Cancer server. Allele highlighted in bold has been predicted to show Cancer-associated SNPs.

| **Gene** | **SNP** | **Mutation** | **PhD-SNP** | **Pmut** | | **Mutpred** | | | **Dr Cancer** | **Fathmm** | **Inference** |
| --- | --- | --- | --- | --- | --- | --- | --- | --- | --- | --- | --- |
|  |  |  |  | **Score** | **Predictions** | **g score** | **P score** | **Molecular changes** |  |  |  |
| **Aurora A** | rs146034224 | P381L | Disease | 0.9355 | Pathological | 0.760 | 0.2106 | Loss of glycosylation at T384 | Cancer | Neutral | Actionable hypothesis |
|  | **rs11539196** | **G325W** | **Disease** | **0.8377** | **Pathological** | **0.947** | **0.0128** | **Gain of catalytic residue at L323** | **Cancer** | **Cancer** | **Confident hypothesis** |
|  | rs45483697 | G198S | Neutral | 0.4515 | Neutral | 0.635 | 0.2336 | Loss of catalytic residue at G198 | Neutral | Cancer | Very Low Confidence |
|  | rs45520831 | R179K | Neutral | 0.1215 | Neutral | 0.626 | 0.017 | Gain of ubiquitination at R179 | Neutral | Neutral | Actionable hypothesis |
|  | rs188825988 | R24C | Disease | 0.8678 | Pathological | 0.258 | 0.0564 | Gain of ubiquitination at K23 | Cancer | Neutral | Very Low Confidence |
| **Aurora B** | rs148715809 | P325S | Neutral | 0.7659 | Pathological | 0.679 | 0.0563 | Gain of MoRF binding | Neutral | Neutral | Very Low Confidence |
|  | rs146334050 | I304T | Neutral | 0.7025 | Pathological | 0.886 | 0.0067 | Loss of stability | Neutral | Neutral | Confident Hypothesis |
|  | rs140224531 | R284C | Neutral | 0.8364 | Pathological | 0.653 | 0.0461 | Gain of ubiquitination at K287 | Neutral | Neutral | Actionable hypothesis |
|  | rs149651741 | G212R | Disease | 0.6926 | Pathological | 0.671 | 0.0477 | Gain of sheet | Cancer | Cancer | Actionable hypothesis |
|  | rs146905713 | E204D | Disease | 0.0386 | Neutral | 0.777 | 0.0477 | Gain of sheet | Cancer | Cancer | Confident hypothesis |
|  | rs55871613 | T179M | Neutral | 0.4040 | Neutral | 0.436 | 0.0394 | Loss of glycosylation at T179 | Neutral | Neutral | Very low confidence |
|  | rs199630207 | E174K | Disease | 0.6532 | Pathological | 0.748 | 0.0138 | Gain of ubiquitination at E174 | Cancer | Cancer | Actionable hypothesis |
|  | rs147097910 | A157T | Neutral | 0.0828 | Neutral | 0.748 | 0.0534 | Gain of phosphorylation at A157 | Neutral | Neutral | Very low confidence |
|  | rs150216235 | V103M | Neutral | 0.2444 | Neutral | 0.802 | 0.1535 | Gain of methylation at K106 | Neutral | Neutral | Very low confidence |
|  | rs184713921 | R95Q | Disease | 0.5915 | Pathological | 0.609 | 0.0262 | Loss of MoRF binding | Cancer | Neutral | Actionable hypothesis |
| **Aurora C** | rs137858773 | R28H | Disease | 0.2955 | Neutral | 0.600 | 0.0713 | Loss of MoRF binding | Cancer | Cancer | Very low confidence |
|  | rs148631645 | I60T | Disease | 0.3218 | Neutral | 0.604 | 0.0295 | Loss of stability | Neutral | Neutral | Actionable hypothesis |
|  | rs45555141 | R86H | Neutral | 0.2767 | Neutral | 0.764 | 0.033 | Loss of MoRF binding | Neutral | Cancer | Actionable hypothesis |
|  | rs45623632 | Y90C | Neutral | 0.6906 | Pathological | 0.710 | 0.013 | Loss of stability | Neutral | Neutral | Actionable hypothesis |
|  | rs199855150 | H92R | Neutral | 0.1666 | Neutral | 0.538 | 0.0098 | Gain of MoRF binding | Neutral | Neutral | Actionable hypothesis |
|  | rs45503793 | T126M | Neutral | 0.4305 | Neutral | 0.440 | 0.0716 | Loss of phosphorylation at T126 | Neutral | Neutral | Very low confidence |
|  | rs45527835 | D236Y | Disease | 0.3598 | Neutral | 0.511 | 0.0344 | Gain of MoRF binding | Cancer | Neutral | Actionable hypothesis |
